# Accuracy assessment of removable partial denture frameworks fabricated by selective laser melting using two different workflows: A cross-over clinical study

**DOI:** 10.1186/s12903-025-06214-9

**Published:** 2025-05-28

**Authors:** Mohamed Elgamal, Abdallah Mohammed Ibrahim, Basem Tarek Fadl, Nourhan Ahmed Ragheb

**Affiliations:** 1 Department of Removable Prosthodontics, Faculty of Dentistry, Mansoura University, Eldakahlia, Egypt; 2 Department of Removable Prosthodontics, Faculty of Dentistry, Horus University, Damietta, Egypt; 3 Department of Removable prosthodontics, Faculty of Dentistry, Kafrelsheikh University, Kafrelsheikh, Egypt

**Keywords:** Selective laser melting, Removable partial denture accuracy, Fully-digital workflow, Combined analog-digital workflow

## Abstract

**Background:**

Rapid advancements in computer-aided design and computer-aided manufacturing (CAD-CAM) have opened new pathways in the fabrication of removable partial dentures (RPDs). Digital impression serves as the first step of CAD-CAM technique, which includes two methods for data acquisition: direct intraoral scanning or indirect extraoral scanning. RPD frameworks may be considered the definitive test of accuracy for a digital workflow. This cross-over clinical study aimed to evaluate the overall accuracy of various parts for mandibular metallic RPD frameworks made by selective laser melting (SLM) using fully-digital versus combined analog-digital workflows.

**Methods:**

This study was carried out on 24 participants with mandibular Kennedy class I arches. Each participant received two RPDs frameworks, one fabricated using a combined analog-digital workflow (Group I) and the other using a fully- digital workflow (Group II). In Group I, the analog steps involved taking physical impressions and creating stone casts followed by scanning the casts with a laboratory scanner to create virtual casts. In Group II, a definitive scan STL file was created using an intraoral digital scanner. Both groups used 3Shape software for digital design of the RPD framework, ensuring consistency by using the same design, and subsequent fabrication using SLM. To evaluate the accuracy, STL data analysis was performed through intra-oral digital superimposition evaluation, a color map was assessed and overall accuracy and the misfit (distance between each framework component and the reference intra-oral scan STL file) were measured at rest, proximal plate, lingual plate and I-bar retentive clasp terminal areas. The Paired t-test was utilized for statistical analysis of the data.

**Results:**

In Group I: combined analog-digital workflow color map assessment revealed more gaps particularly in the lingual plate and proximal plate areas, whereas Group II: fully-digital workflow showed better accuracy. Regarding the overall accuracy in the rest, proximal plate, lingual plate, and I-bar clasp retentive terminal, Group II was significantly superior to Group I (*P* = 0.0125, 0.0019, < 0.001, < 0.001, and 0.0119, respectively).

**Conclusions:**

Within the limitations of this study, both workflows resulted in frameworks with clinically acceptable accuracy. However, SLM RPD frameworks fabricated using the fully-digital workflow showed superior accuracy in key areas such as the rest, proximal plate, lingual plate, and I-bar clasp retentive terminal areas, when compared to the combined analog-digital workflow.

**Trial registration:**

Retrospectively registered (NCT06412159) 03/05/2024.

## Background

There are several treatment options to restore partially edentulous arches, including tooth and implants-supported restorations [1]. Despite the high success rates of implant treatments, removable partial dentures (RPDs) remain the treatment of choice for a large number of patients especially when patients have medical problems that may be associated with absolute contraindications to implant rehabilitation like recent myocardial infarction, valvular prosthesis surgery, immunosuppression, bleeding issues, active treatment of malignancy, drug abuse, psychiatric illness, as well as intravenous bisphosphonate or relative contraindications like adolescence, aging, osteoporosis, smoking, diabetes, radiation therapy and cardiovascular disease [2, 3].

RPDs are conventionally made using the lost wax method, which can result in an ill-fitting casting due to refractory cast distortion and wax pattern distortion [4]. Digital technologies have been recently utilized to fabricate RPDs aiming to minimize human error, save time, and reduce material usage [5, 6]. Additionally, recent data indicates that, in comparison to conventionally manufactured RPDs, RPDs fabricated with digital technology resulted in higher patient satisfaction [7].

Recent developments in computer-aided design and computer-aided manufacturing (CAD-CAM) have opened up new possibilities for fabricating RPD frameworks through both additive and subtractive methods directly from digital designs. Selective laser melting (SLM) is among ten additive manufacturing 3-dimensional (3D) printing techniques [8]. SLM is a process that uses high-power lasers to melt metal powders, fusing the powder particles into a solid layer. Titanium and cobalt-chromium alloy (Co- Cr) can be printed using this method to construct RPD frameworks [9, 10]. RPD frameworks fabricated using the SLM technology was found to yield clinically satisfactory results [11]. Moreover, it has been reported that SLM Co-Cr alloy frameworks exhibited superior mechanical and microstructural qualities compared to cast or milled RPD frameworks [12]. Furthermore, the alloy would be more resistant to distortion due to this manufacturing technique, which could result in a more advantageous distribution of occlusal force across the remaining teeth or supporting tissues [13].

Creating a digital model of the intraoral hard and soft tissues is part of the standard digital workflow. For a fully-digital workflow, it can be done directly by using an intraoral scanner. Alternatively, for a combined analog-digital workflow, it can be done by scanning the stone cast itself using a laboratory scanner [14, 15]. Regarding the accuracy of intraoral digital scans of partially edentulous arches, the literature is inconsistent. For teeth in partially dentate arches the literature generally indicates that digital scans are as accurate as conventional impressions [16]. Moreover, recent research has demonstrated that digital scans incorporating intra-oral scans (IOSs) are significantly more accurate than impressions made of silicone for partially edentulous arches [16–18].

Conversely, some research has found that scanning edentulous tissue is more challenging and less accurate than scanning hard tissue [19, 20]. It should be noted that soft tissues can depress up to 300 μm below the distal extension of a RPD so, high accuracy is not crucial in edentulous regions, however the potential for soft tissue displacement and the risk of decubitus must be considered [19].

Proper accuracy of RPD frameworks is crucial for the success of such restorations [21]. With the advent of new digital technologies, new assessment methods for the accuracy of an RPD framework are being possible as these techniques can measure 3D discrepancies directly, like digital superimposition, without the need for an intermediary substrate [22]. Additionally, the color mapping features in these new technologies provides important general data about the accuracy and adaptability of these restorations [20, 23, 24].

There is still much debate in the literature regarding the accuracy of fully- digital RPD frameworks compared to combined analog-digital workflows. While digitally created frameworks have shown to meet clinical requirements, the results of accuracy vary between studies due to differences in assessment techniques. However, there has been no research to directly compare the accuracy of fully-digital RPD frameworks with hybrid analog-digital fabrication workflows. The aim of this study was to use STL data analysis and intra-oral digital superimposition evaluation to assess overall accuracy of fully-digital versus combined analog-digital workflows, as well as the accuracy of each component of the RPD framework. The null hypothesis is that there would be no significant differences in the accuracy of RPD frameworks fabricated using the two workflows.

## Materials and methods

### Study design

In this crossover study, each participant received two cobalt chromium SLM RPD frameworks using two different workflows. The standardization of variables that affect results was made possible by this design, as each participant was his own control. The first workflow was a combined analog-digital workflow (group I), while the second was a fully-digital one (group II). The same lab technician used the same machinery and followed the same procedures for fabricating all SLM RPD frameworks. To compare the two frameworks, the following parameters were assessed using intra-oral digital superimposition technology: (1) overall accuracy, and (2) accuracy of specific components such as occlusal rests, proximal plate, lingual plate, and retentive terminal of I-bar clasp.

### Sample size calculation

A computer software program (G*power version 3.1.5; Heinrich Heine University Düsseldorf) was used to calculate the sample size through a power analysis. The computations were based on data from a previous study [25]. This study found a significant difference in accuracy between the major connectors of RPDs obtained from extraoral and intraoral digital impressions. The sample size was determined to be 24 participants [Pooled SD = 8.52, Effect size = 0.42, type I error (α) = 0.05, type II error (β) = 0.80]. The Ethics Committee of the faculty reviewed and approved the study (KFSIRB200-130) which was also registered on Clinical Trials.gov (NCT06412159), and complied with the Consolidated Standards of Reporting Trials (CONSORT) recommendations for clinical trials. Participants were informed about the study and provided their consent by signing an informed consent form.

#### Inclusion criteria

**Fig. 1:**
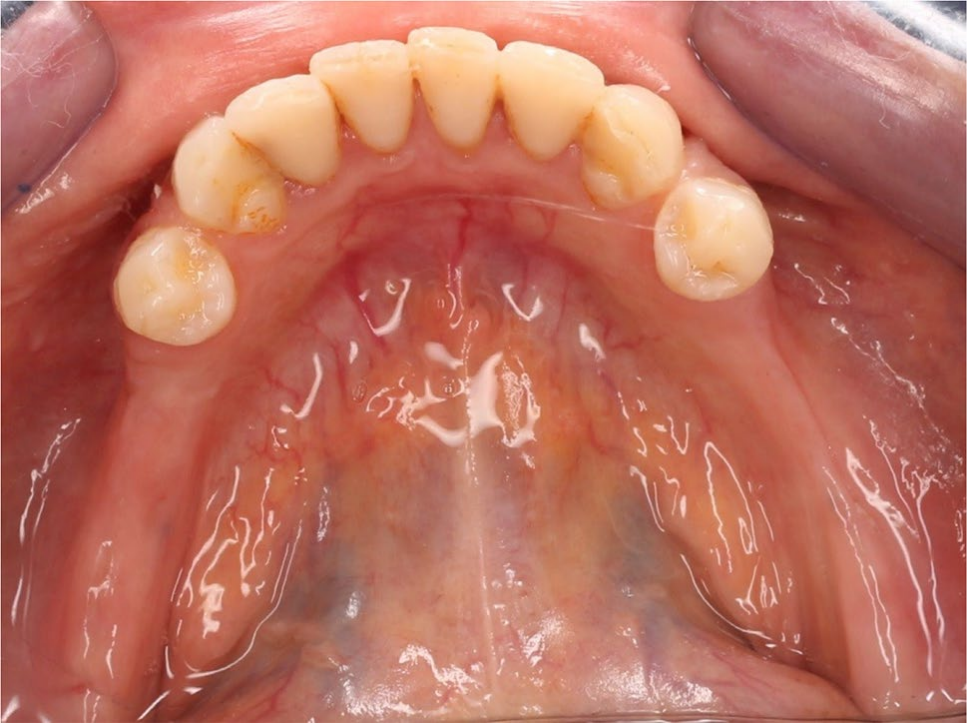
Mandibular arch with bilateral distal extension areas with the first premolars as the last standing abutments

Twenty-four partially edentulous participants between the ages of 50 and 65 were included from the outpatient clinic of the Prosthodontic Department at the Faculty of Dentistry. The inclusion criteria for this study were as follows: Mandibular arches with bilateral distal extension areas (classified as Class I according to the Kennedy classification) Fig. 1, healthy and firm mucosa covering the ridges, remaining periodontally healthy teeth with no mobility including the first premolars on both sides which was opposed by either maxillary natural teeth or fixed restorations. Vestibular depth had to be sufficient to allow the I-bar clasp to be kept 3 mm away from the gingival margin.

### Fabrication of RPD frameworks

For all participants, cingulum rest seats on canines, mesial saucer shaped occlusal rests and distal guiding planes of 1.5 mm height were prepared in the mandibular first premolars.

For group I (combined analog-digital workflow), mandibular primary irreversible hydrocolloid (C A 37 Superior Pink, Cavex, Holland bv) impression was recorded and poured in dental stone (Moldano, Bayer Dental D-5090 Lever Kusen). Custom tray was fabricated using light-cured acrylic material (Vertex Dental, Soesterberg, Netherlands) with a 3 mm spacer and four equally distributed stops. In the edentulous areas, border molding was accomplished using green modeling plastic impression compound (Green Stick Compound; Kerr Corp). The intaglio surface was coated with tray adhesive (VPS Tray adhesive, Kerr Corporation, Orange, CA, USA) and a physical definitive mandibular medium body polyvinyl siloxane (Zhermack INC., 45021 Badia, Polesine, Rovigo, Italy) impression was made and a stone cast was poured using Type IV stone (Silky-Rock; Whip Mix Corp). A laboratory scanner (3-shape D900 digital scanner) was then used to scan the cast and generate a virtual 3D master model.

For group II (fully-digital workflow), a direct digital scan was made using an IOS (TRIOS 3; 3Shape) to create a definitive scan, that will serve as the reference scan STL file (RS-STL). To ensure high-quality IOS, specific precautions were taken to control moisture effectively by suction devices, or air-drying techniques to ensure the scanning area were dry and free of moisture before and during the scan. Special attention was given to dry the rest seats effectively before scanning by a gentle blotting with sterile gauze, cleaning the surfaces and scan powder was applied to improve the contrast. The scanning strategy (T-R) described by Chang et al. [26] was followed, starting from the dentate area and moving to the edentulous region. The edentulous region posteriorly was then scanned in a zig-zag pattern after the dentate area, while keeping a distance of 10.0 mm between the tip of the scanner and the area to be recorded, as recommended by Rotar et al. [27] for achieving the most accurate results.

**Fig. 2:**
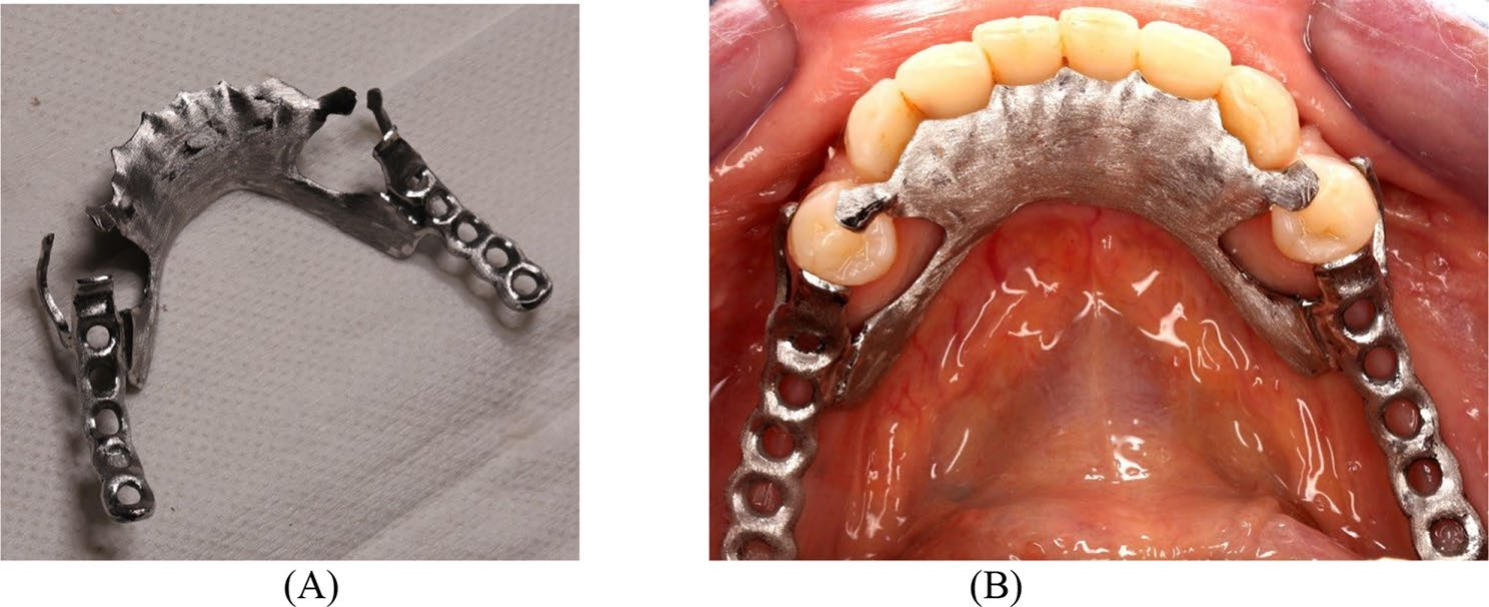
SLM framework. **A**-Extra-oral after finishing and polishing steps. **B**-Intra-oral try in

The same participant STL files were uploaded to the CAD software (3Shape Dental System; 3 Shape A/S, Copenhagen, Denmark) for both workflows. This allowed for the digital design of the RPD framework (the same design was used for consistency), which included a lingual plate mandibular major connector, meshworks on both edentulous ridges, a distal proximal plate, a mesial occlusal rest and I-bar retentive clasp arms on the mandibular first premolars. That was followed by exporting and sending the digital RPD framework designs to be SLM (NCL-M2150X; Nanjing Chamlion Laser Technology Co., Nanjing, China) formed in Co-Cr alloy (EOS Cobalt Chrome SP2; EOS GmbH). After printing, SLM frameworks were finished by sandblasting and diamond burs and subsequently polished with silicone rubber cups before being tried in the participant’s mouth. Finishing and polishing were done by the same dental technician (Fig. 2A).

During try in a structured clinical assessment was followed, where RPD frameworks was inspected clinically by mirror and explored, when rest seats are correctly seated, the metallic parts adapt properly on the teeth, and the soft tissues are not impinged on by any part of the RPD considered a well fitted. Then the framworks seating, retention, and stability was assessed in the oral cavity to ensure that no significant discrepancies were present (Fig. 2B).

### Assessment using the 3 scans technique

The accuracy for each framework in both groups was evaluated using intra-oral digital superimposition, utilizing the 3 scans technique [22, 28].

**Fig. 3:**
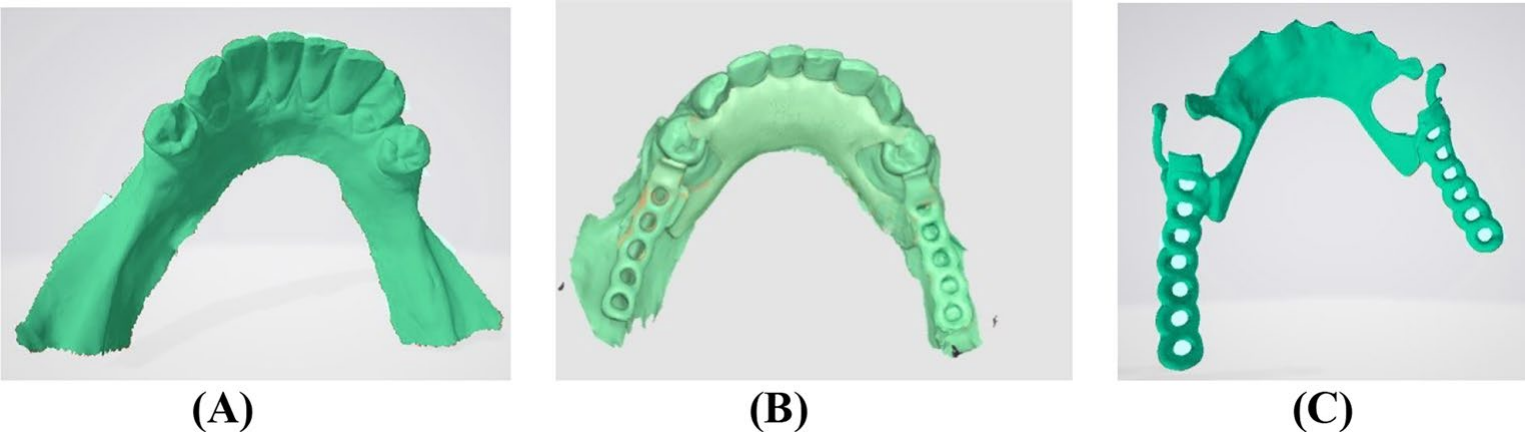
The STL files for the reference scan and the test scan were uploaded into Medit Compare software. **A**- STL file of the intraoral scan without the framework (RS-STL) (1-st scan). **B**- STL file of the intraoral scan with the seated framework (2-nd scan). **C**- STL file of the extraoral scan of the framework alone (TS-STL) (3-rd scan)

First scan: An intraoral scan without the framework (RS-STL). Second scan: An intraoral scan with the framework seated inside the participant’s mouth to help in the future alignment of the scans. Third scan: An extra oral scan of the framework alone using intraoral scanner, both polished and fitting surfaces of the framework were captured outside the participant’s mouth producing (TS-STL) file (Fig. 3A, B & C). During the third scan the framework was fixed to the scanning table using two pieces of plasticine attached to the lower border of the lingual plate, and the scanning procedure was performed from various angles to obtain both the fitting surface and the polished surface of the framework. Furthermore, reflective surfaces of the RPD frameworks were managed using scan powder to minimize scanning errors due to surface reflectivity. An even, thin layer of powder was applied to all metal objects, furthermore excess powder or residue left carefully wiped away to avoid any inaccuracies on the scanning process.

**Fig. 4:**
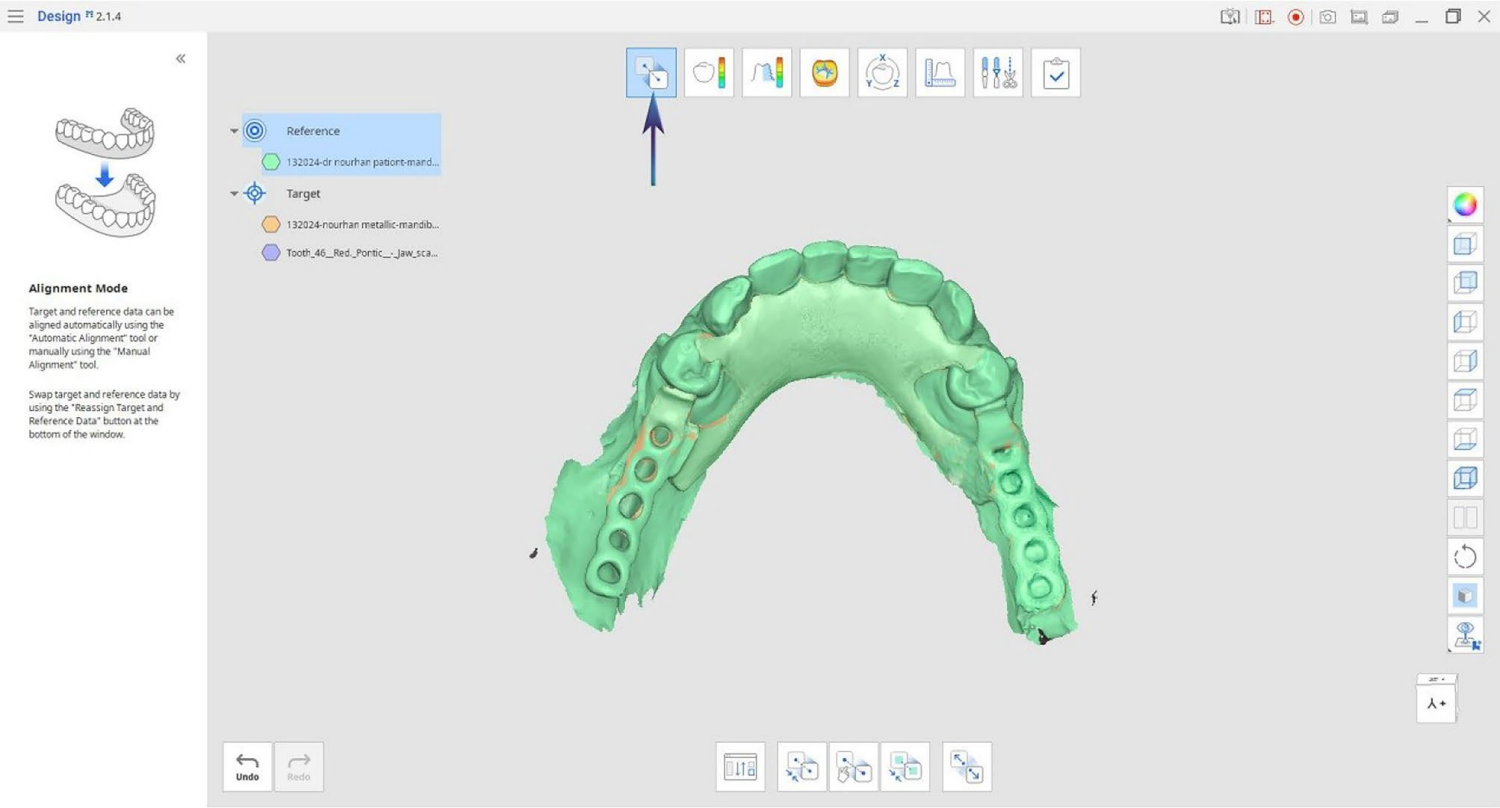
Automatic alignment was used to align STL files. The arrow indicates the tool to be used for the alignment of the reference and test scan STL files

The TS-STL file was superimposed over the RS-STL file using the automatic alignment tool in the software (Medit Compare v1.1.1.61, Medit, Seoul, Republic of Korea) (Fig. 4). Once the three scans were accurately aligned, the second scan was excluded and the gaps were measured vertically between the first and third scan to conduct the accuracy assessments [22].

### Color map evaluation

**Fig. 5:**
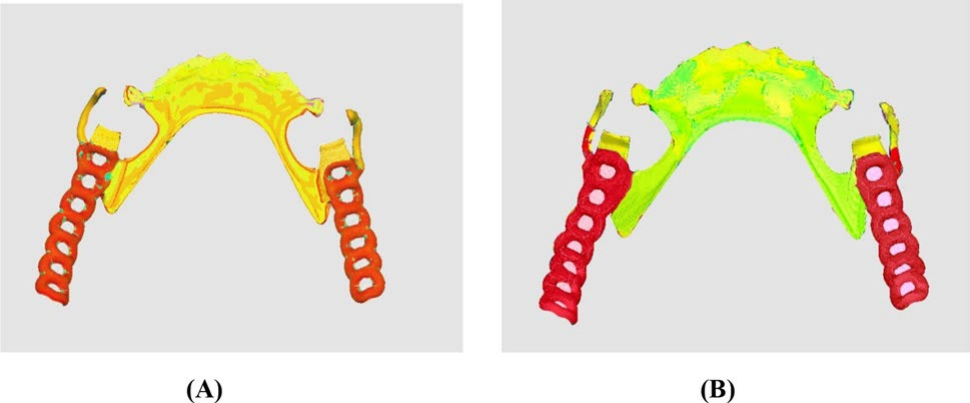
Color maps representative samples of the accuracy generated by the superimposition of TS-STL file over the RS-STL. (**A**) framework for Group I: combined analog-digital workflow. (**B**) framework for Group II: fully-digital workflow

The color maps in Fig. 5 show the differences in surface matching between the frameworks from each group and the reference scan. A close accuracy between the framework and the tooth structure would result in a green color, while blue areas indicate excessive contacts, yellow to orange areas indicate gaps but within acceptable accuracy, and red areas mean more gaps between the framework and the RS-STL file indicate an unacceptable accuracy.

### Accuracy measurements

**Fig. 6:**
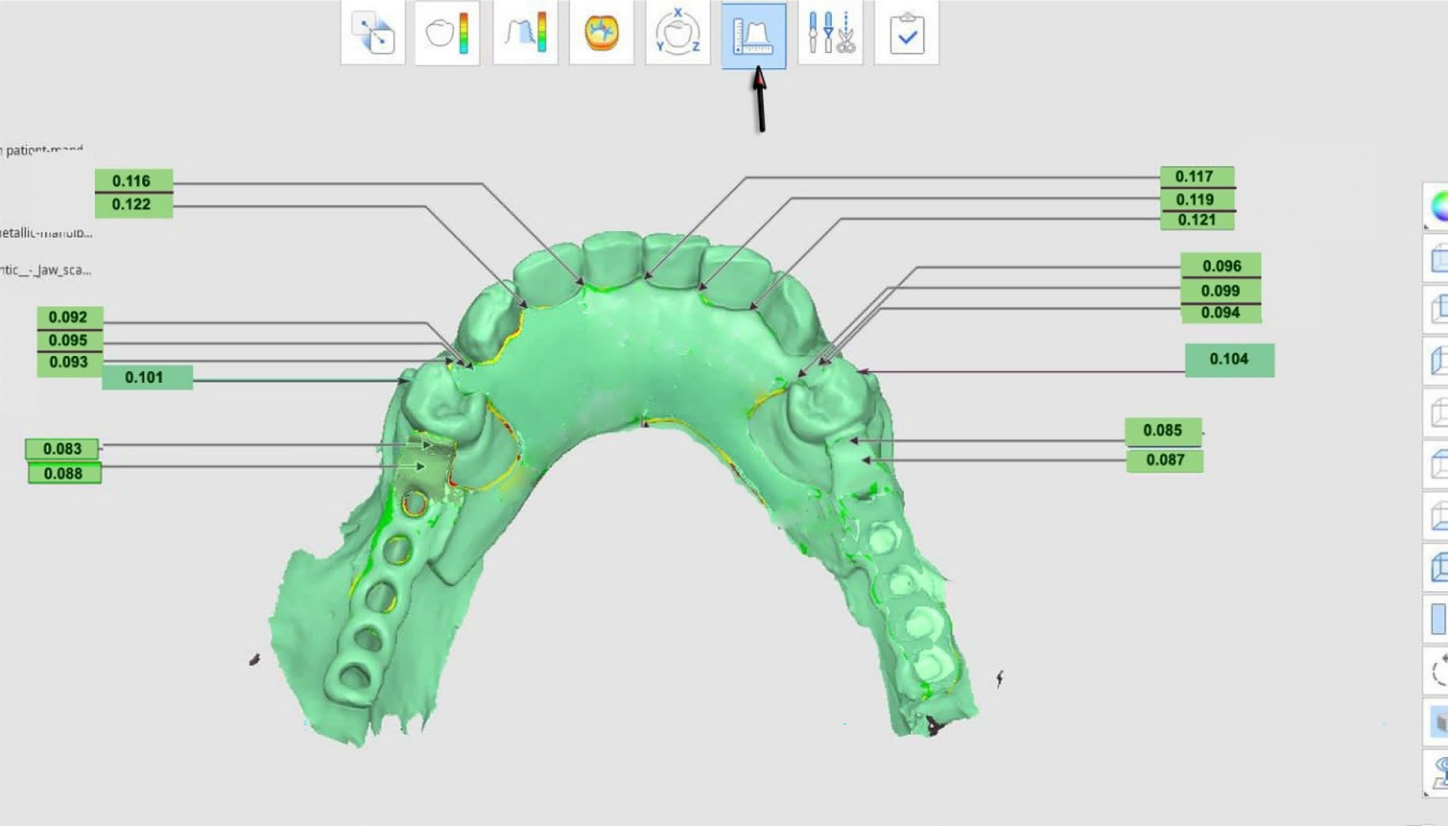
After digital superimposition, Medit Compare automatically calculates the accuracy measurement of specific areas throughout the entire framework and shows the distance between the RS-STL file made with an IOS and the TS -STL file. The arrow indicates the tool to be used for the measurement of distance between the two STL files

**Fig. 7:**
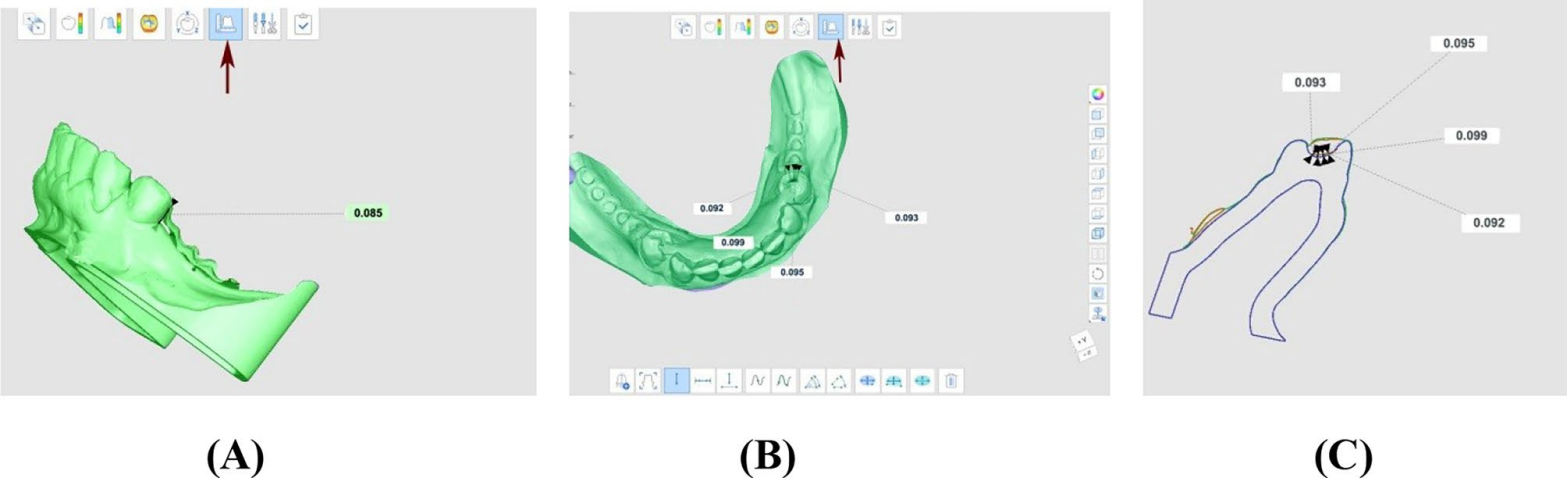
Medit Compare software used for gap measurement between the framework and the teeth, **A**- At proximal plate area. **B**- At rest area. **C**- 2D cross sectional gap measurement between the rest and rest seat area

**Fig. 8:**
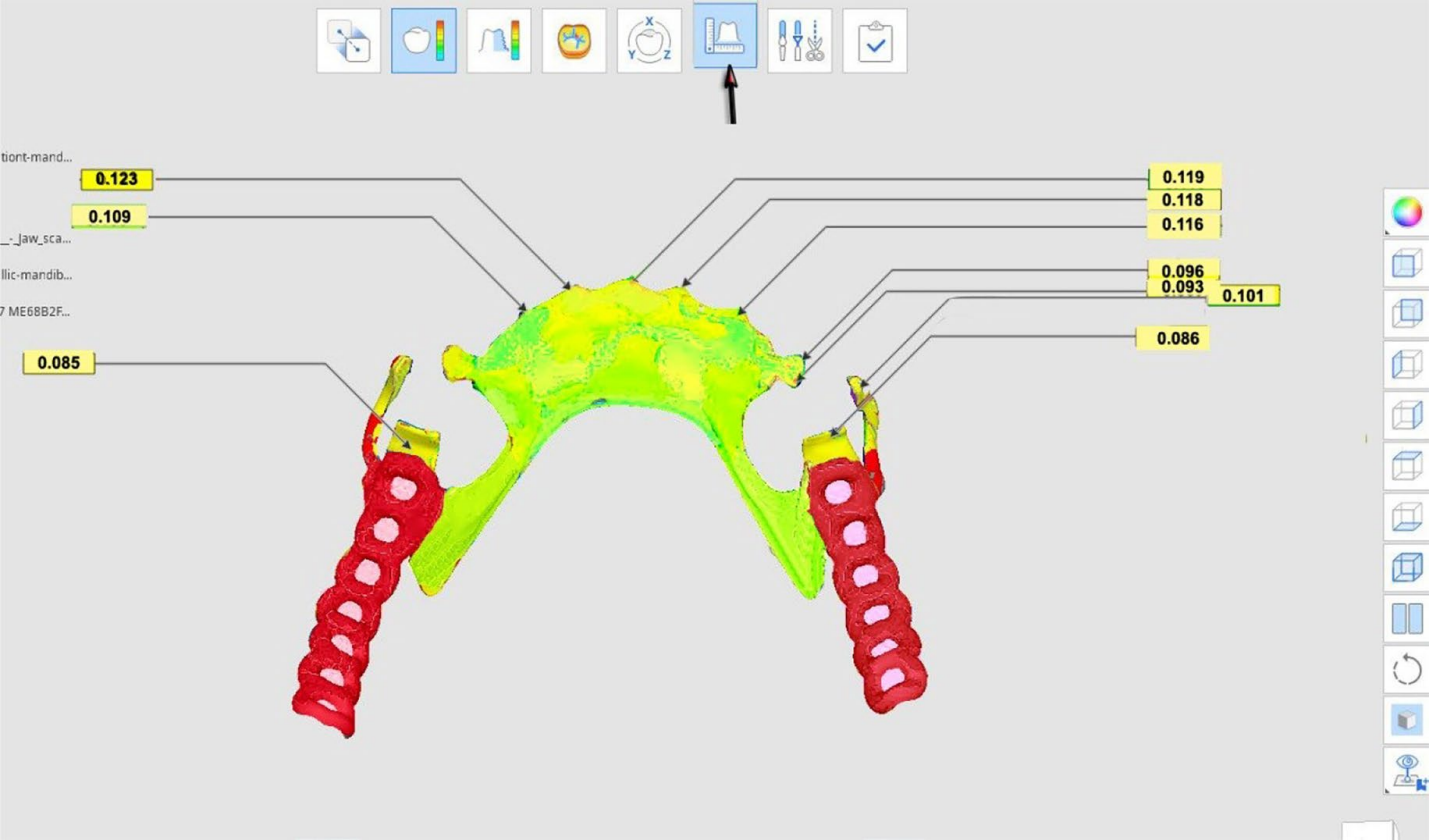
Digital superimposition and measurement the accuracy of specific areas throughout the whole framework and shows the distance between the RS-STL file made with an IOS and the TS- STL file. The arrow indicates the tool to be used for the measurement of distance between the two STL files

The accuracy of measurement was evaluated using a non-metrology grade Medit software program. Both the overall accuracy as well as the misfit in certain areas (the rest/rest seat areas of premolars using the cross-sectional measurements, the right and left guide plane/plate area, the superior border of the lingual plate area, and retentive terminal of I-bar clasp were assessed (Figs. 6, 7 and 8).

All measurements were conducted by the same investigator and repeated five times to ensure reliability. The average measurement error was found to be approximately 0.002 mm. In evaluating the misfit, we considered a distance of 0 to 0.05 mm to indicate close contact with no gap, and a distance of 0.05 to 0.31 mm to be an acceptable accuracy from a clinical standpoint. All gaps greater than 0.31 mm would, therefore, be deemed clinically unacceptable [29–31].

### Statistical analysis

Data were tabulated and statistical analysis was performed on the data using SPSS software (SPSS v25, Inc., Chicago, IL). The data are displayed as the mean and standard deviation (SD), and Paired t-test was employed. The overall accuracy as well as the accuracy of each area was compared in this analysis. Statistical significance was determined by considering *P* < 0.05.

## Results

### Color map evaluation

Regarding the visual analysis of the color map, the results showed a higher amount of spaced areas (indicated by the yellow color) in Group I: combined analog-digital workflow, particularly in the proximal plate and lingual plate areas. This suggests that there was more space between the scanned fitting surface and the RS-STL file, indicating that the frameworks were not fully adapted on the tissues intraorally. In contrast, Group II: fully-digital workflow displayed a more distribution of green color on the map, indicating that the frameworks were more adapted on the tissues intraorally.

### Accuracy measurements

**Fig. 9:**
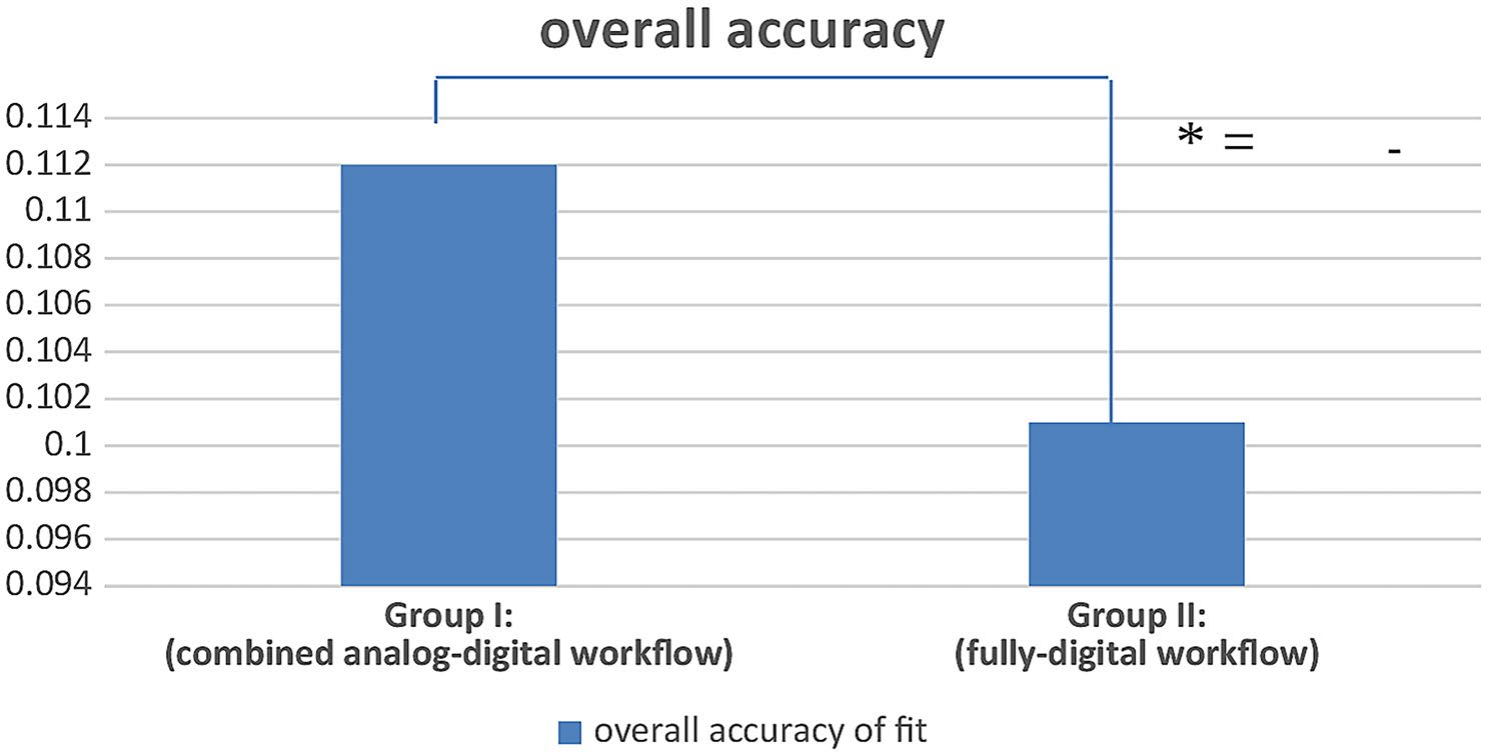
Showing measured overall mean accuracy (mm) of all frameworks for combined and fully-digital workflows

In terms of evaluating the accuracy, the distances between each framework and the reference scan were measured in millimeters (mm) for each framework in each group. The overall mean distance (gap) between the frameworks and the reference scans was significantly greater in Group I than in Group II as (Table 1; Fig. 9).

**Table 1:** Comparison of the overall mean accuracy (mm) for all frameworks of each group

Component	Group I: (combined analog-digital workflow) (Mean ± SD)	Group II: (fully-digital workflow) (Mean ± SD)	t-test	*P* value
Overall mean accuracy	0.112 ± 0.018	0.101 ± 0.013	2.5683	0.0125*

(Paired t-test, *P* < 0.05) *P is significant at 5% level

**Fig. 10:**
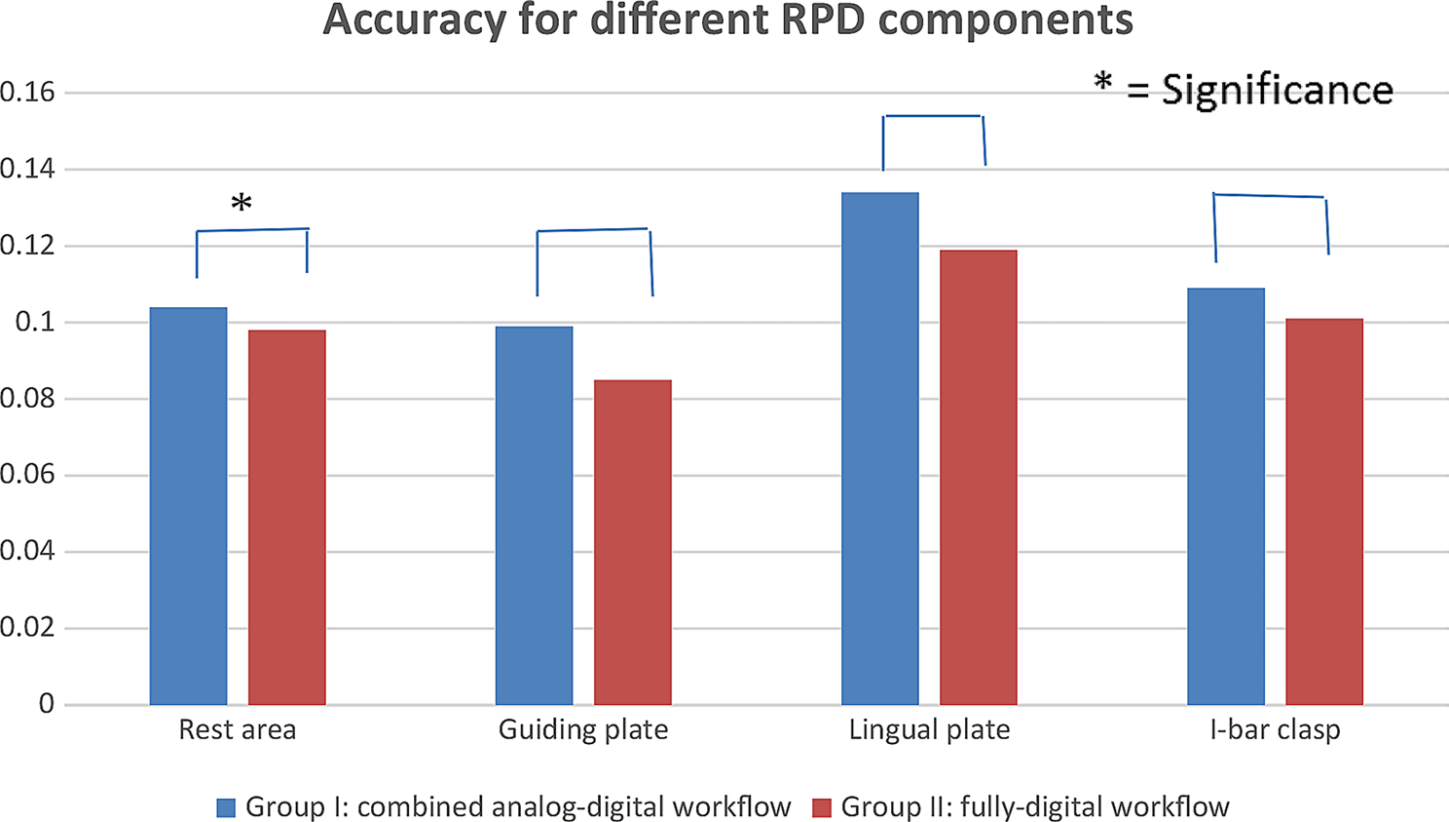
Showing measured mean accuracy (mm) under occlusal rest, proximal plate, lingual plate major connector and terminal end of I-bar clasp retentive arm for combined and fully-digital workflows

Table 2 presents additional measurements taken between the frameworks and the reference scan for each participant in specific areas, including occlusal rests, proximal plates, the lingual plate major connector and I-bar clasp arm terminals. The mean distance (gap) between the frameworks and the reference scans was significantly higher in Group I than in Group II for all specified areas: the rest areas, the proximal plates, the lingual plate area, as well as the I-bar clasp arm terminal area (Table 2; Fig. 10).

**Table 2:** Comparison of mean accuracy (mm) under occlusal rest, proximal plate, lingual plate major connector and terminal end of I-bar clasp retentive arm for each group

Component	Group I: (combined analog-digital workflow) (Mean ± SD)	Group II: (fully-digital workflow) (Mean ± SD)	t-test	P value
Rest area accuracy	0.104 ± 0.007	0.098 ± 0.006	3.7974	0.0019*
Proximal plate area accuracy	0.099 ± 0.008	0.085 ± 0.008	6.8560	*<* 0.001*
Lingual plate area accuracy	0.134 ± 0.012	0.119 ± 0.007	5.6748	*<* 0.001*
Terminal end of I-bar clasp retentive arm	0.109 ± 0.013	0.101 ± 0.009	2.5974	0.0119*

(Paired t-test, *P* < 0.05) *P is significant at 5% level

## Discussion

In this study, the null hypothesis was rejected as the frameworks constructed following the fully-digital workflow exhibited significantly better accuracy values than those fabricated following the combined analog-digital workflow.

RPDs may be considered the definitive assessment test of accuracy for a digital workflow as the fabrication of the RPD framework necessitates precise capture of both hard and soft tissue anatomy. Close adaptation and the relationship between the different RPD components and their supporting abutment teeth are critical not only for their effectiveness, but more importantly to minimize any adverse effects on the supporting abutments and avoid any patient discomfort. Moreover, any gaps between the prosthesis and mucosa may create possible plaque-retentive food traps that may promote tissue inflammation [22].

In the current study, visual assessment of the color map was used to evaluate the accuracy to determine the clinical value of two different workflows for fabricating SLM RPD frameworks. Overall, the mean accuracy results were considered acceptable for both workflows, as all recorded values were less than 0.31 mm [29–31]. This finding is consistent with the findings of previous studies. According to the study conducted by Gan et al. RPD major connectors fabricated using both the fully digital and the combined- conventional workflows demonstrated clinically acceptable adaptation [25]. Kattadiyil et al. [17] effectively created an RPD through the use of a digital workflow and it was clinically acceptable. Suzuki et al. [32] illustrated how an RPD made using TRIOS 3 to obtain the digital impression could achieve excellent adaptive accuracy. Schimmel et al. [33] pointed out that whatever the degree of experience of the clinician, newer versions of IOS scanners might be appropriate for the creation of clinically acceptable RPD frameworks. Despite the limited number of clinical trials about the use of IOS and digital workflow in the fabrication of RPD frameworks, these studies justify our results as they reinforce the claim that the accuracy of RPDs fabricated using a fully digital workflow was acceptable [34, 35].

It is worth mentioning however, that there was a significant difference in the accuracy between the two workflows. The overall mean accuracy values for the combined analog-digital workflow and the fully-digital workflow were 0.112 ± 0.018 mm and 0.101 ± 0.013 mm, respectively. This indicates that the fully-digital workflow resulted in frameworks with superior accuracy. In addition to the mean overall accuracy, the accuracy of the individually tested RPD framework components was also superior in the fully-digital workflow group as compared to the combined analog-digital workflow group. This may be attributed to the fact that the process of transitioning between analog and digital workflows can lead to a buildup of errors at each step [22, 23]. The weakest point in combined workflows for RPD fabrication is probably during the impression stage. One possible explanation for this is the accumulation of mistakes during the physical impression, pouring it into the stone, and scanning it into a digital cast. These three steps inherently contain errors that could result in noticeable discrepancies in a rigid RPD framework [36].

In line with our research, a clinical study conducted by Tregerman et al. [36] evaluated the effectiveness of three different fabrication methods for RPD frameworks: analog, combined analog-digital and fully-digital where each participant received three RPD frameworks using each of the three techniques. Their assessed was based on a subjective clinical evaluation using a yes/no questionnaire survey, not objective assessments that depended on actual accuracy measurements as used in the current study. However, despite this difference, their subjective results were consistent with the objective results of the current study, as the fully-digital workflow (including IOS, designing, and SLM printing) was significantly more effective than the other two workflows including the combined analogue-digital workflow. The authors attributed the high performance of the fully digital workflow to the greater accuracy of intraoral scanning compared to traditional impressions [36].

Tasaka et al. [23] also confirmed our results through an in vitro study comparing the accuracy of castable 3D-printed resin pattern frameworks (a combination of analog and digital workflow) and SLS metal printed frameworks (a fully-digital workflow). The study reported a significant difference in accuracy at the rests, proximal plates, connectors, and clasp arms, with more discrepancy in the combined workflow compared to the fully digital SLS printed group. Furthermore, recent in vitro studies, have suggested that completely digitally fabricated RPD frameworks have superior accuracy compared to purely analog or combined analog-digital workflows [23, 37, 38]. However, these in vitro studies have several limitations, including the lack of consideration for environmental factors such as saliva, blood, and soft tissue changes.

On the contrary, a study conducted by Gan et al. [25] compared the adaptation of RPD major connectors fabricated from intraoral and extraoral digital impressions using printed resin frameworks. They found that the adaptation of maxillary major connectors fabricated from intraoral digital impressions was inferior to those fabricated from extraoral digital impressions. This difference may be related to the different assessment methodology they used. They assessed adaptation intraorally and palatally only using light body silicone impression materials. Another in vitro study conducted by Korkes et al. [39] on a Kennedy class III modification 1 partially edentulous mandibular arch cast, aimed to evaluate the seating accuracy of RPD frameworks fabricated using two digital workflows. Unlike our study, the marginal gap was assessed using an optical traveling microscope and the results showed that the combined analog-digital workflow group had the most superior accuracy, followed by the fully digital workflow group, with the conventional workflow group showing the least accuracy. The difference between their study and the current study, maybe related to the Kennedy Class and complexity of the frameworks. Frameworks in the current study had different dimensions with less components and a simpler design as there were no modification areas.

In this study, the observed overall mean deviation value between the two groups was 0.011 mm which is higher than the lowest value of discrepancy 0.005 mm reported in a previous study by Soltanzadeh et al. [31]. Although there was a statistically significant difference in the fit accuracy of SLM RPD frameworks produced by both workflows, the deviation difference observed in this study has no clinical importance, as supported by a previous study, who stated that ``up to 0.12 mm difference in overall mean vertical gaps may have no clinical significance due to the oral resiliency of the soft tissues `` [40].

One limitation of this study is that it only included cases with a mandibular Kennedy class I design. Future studies could expand the scope by including more complex cases, such as Kennedy class III or Class II designs with modification areas as they will include more RPD components, as well as cases with different edentulous span lengths. This could provide a more comprehensive understanding of accuracy of RPD frameworks of different designs. Another limitation is that the study measured the accuracy at only four locations of the RPD framework. This may not provide a comprehensive assessment of the accuracy in all areas including tissue-related areas. We recommend future studies evaluating soft tissue contact that could provide several advantages in assessing the overall accuracy of an RPD framework and would provide a more holistic assessment of framework accuracy.

Other limitation of our study that direct accuracy validation of Medit Compare (non metrology-grade software) used in this specific workflow against other metrology-grade software was not performed. Additionally, it is important to note that the resolution of the scanner used can have an influential impact, potentially leading to measurement errors [41]. To overcome limitation of using single reference scan (RS-STL) in accuracy assessments, we recommend future studies using a cross-over design such as comparing the intraoral scan with the scans from the combined analog-digital workflow or superimposing the scanned RPDs from both methods on laboratory scans of conventional casts that could provide additional insights, help to minimize the potential bias introduced by using the same intraoral scan (RS-STL) as reference for both the accuracy evaluation and the fully-digital workflow. Therefore, it is important to consider these factors when interpreting the current results.

## Conclusions

Within the limitations of this study, it was found that both workflows resulted in frameworks with clinically acceptable accuracy. However, SLM RPD frameworks fabricated using the fully-digital workflow showed superior accuracy in key areas such as the rest, proximal plate, lingual plate, and I-bar clasp retentive terminal areas, when compared to the combined analog-digital workflow.

## Data Availability

The datasets analyzed and materials used during the current study are available from the corresponding author on reasonable request and will be sent.

## Abbreviations

SLM: Selective laser melting
RPD: Removable partial denture
CAD-CAM: Computer-aided design and computer-aided manufacturing
Co- Cr: Cobalt-chromium
IOS: Intra-oral scanner
3D: 3-dimensional
RS-STL: Reference scan STL file
TS-STL: Test scan STL file
mm: Millimeters
SD: Standard deviation
